# Loss of Coupling Distinguishes GJB1 Mutations Associated with CNS Manifestations of CMT1X from Those Without CNS Manifestations

**DOI:** 10.1038/srep40166

**Published:** 2017-01-10

**Authors:** Charles K. Abrams, Mikhail Goman, Sarah Wong, Steven S. Scherer, Kleopas A. Kleopa, Alejandro Peinado, Mona M. Freidin

**Affiliations:** 1 Department of Neurology and Rehabilitation, College of Medicine, University of Illinois at Chicago, Chicago IL, USA; 2 Department of Neurology, SUNY Downstate, Brooklyn, NY, USA; 3 Department of Neurology, The Perelman School of Medicine at the University of Pennsylvania, Philadelphia, PA, USA; 4 Neurology Clinics and Neuroscience Laboratory, Cyprus Institute for Neurology and Genetics, Nicosia, Cyprus

## Abstract

CMT1X, an X-linked inherited neuropathy, is caused by mutations in *GJB1*, which codes for Cx32, a gap junction protein expressed by Schwann cells and oligodendrocytes. Many *GJB1* mutations cause central nervous system (CNS) abnormality in males, including stable subclinical signs and, less often, short-duration episodes characterized by motor difficulties and altered consciousness. However, some mutations have no apparent CNS effects. What distinguishes mutations with and without CNS manifestations has been unclear. Here we studied a total of 14 Cx32 mutations, 10 of which are associated with florid episodic CNS clinical syndromes in addition to peripheral neuropathy. The other 4 mutations exhibit neuropathy without clinical or subclinical CNS abnormalities. These “PNS-only” mutations (Y151C, V181M, R183C and L239I) form gap junction plaques and produce levels of junctional coupling similar to those for wild-type Cx32. In contrast, mutants with CNS manifestations (F51L, E102del, V139M, R142Q, R142W, R164W T55I, R164Q and C168Y) either form no morphological gap junction plaques or, if they do, produce little or no detectable junctional coupling. Thus, PNS and CNS abnormalities may involve different aspects of connexin function.

Charcot-Marie-Tooth disease (CMT) is a group of disorders characterized by exclusive or predominant involvement of the PNS^1^. CMT1X, the X-linked form is caused by mutations in GJB1^2^, which codes for Cx32, a gap junction (GJ) forming protein expressed in both Schwann cells and oligodendrocytes, the myelinating cells of the PNS and CNS, respectively^3^. In myelinating Schwann cells, Cx32 is thought to form a reflexive pathway, connecting adjacent loops of noncompact myelin^3^ and shortening the path between ab- and adaxonal membranes by at least 300-fold^3^,^4^. In the CNS, Cx32 participates in the GJ coupling between oligodendrocytes and astrocytes (O:A coupling; likely mediated by Cx32:Cx30 and Cx47:Cx43 heterotypic channels), and between oligodendrocytes themselves (O:O coupling, likely mediated by Cx32:Cx32 and Cx47:Cx47 homotypic channels^5^,^6^,^7^).

The expression of Cx32 in the CNS is restricted to oligodendrocytes^3^. It has been proposed that disrupted O:A and/or O:O GJ coupling accounts for the presence of subclinical CNS findings in CMT1X– primarily slowed evoked potentials (visual evoked responses, VERs and brainstem auditory evoked responses, BAERs)^8^. In addition there is a growing literature describing static, clinical CNS manifestations of CMT1X such as spasticity and hyperreflexia^9^, ataxia^10^, cognitive impairment^11^, or the presence of a Babinski sign^12^; abnormal static brain MRI findings without mention of clinical correlate have also been reported^13^,^14^. (See Abrams and Freidin^8^ for review and additional references.) Finally, a growing number of patients with otherwise typical CMT1X have been found with an acute, florid syndrome characterized by dysarthria, motor difficulties and variable alterations in level of consciousness^3^,^15^,^16^,^17^,^18^,^19^,^20^,^21^,^22^,^23^,^24^,^25^,^26^,^27^,^28^,^29^,^30^. These clinical phenomena are transient, generally lasting from hours to days, and are accompanied by longer lasting changes in brain MRI imaging, with prominent increased signal in the splenium of the corpus callosum and the deep cortical white matter on diffusion weighted imaging. In this report we refer to mutations causing these acute florid syndromes as PNS + CNS mutations. CMT1X patients with a small number of identified point mutations, specifically *L239I, Y151C, V181M, and R183C* (ref. 31 and G. Nicholson, personal communication), do not have these subclinical CNS abnormalities. We refer to these here as PNS-only mutations and provide evidence that they likely do not disrupt O-O or O-A coupling significantly. For clarity the PNS-only mutations are italicized throughout the manuscript.

Until now only one of the clinically characterized mutations associated with florid CNS symptoms (R75W) has been examined electrophysiologically in mammalian expression systems, where the most reliable characterization can be made using dual whole-cell voltage clamp^32^. However, no systematic examination of the effects of clinically characterized mutations on their ability to form functional gap junctions has been undertaken. For example, the PNS-only mutations mentioned above (i.e. those reported as not being associated with any CNS findings) have not previously been studied with respect to their gap-junction forming ability. In this communication we report on our findings in HeLa and Neuro2a cells regarding the ability of 14 mutant forms of Cx32 to form functional gap junctions; 4 mutants are PNS-only and 10 mutants are associated with episodic and florid CNS clinical syndromes as well. The 10 PNS + CNS mutants studied here, with the R75W mutant previously studied, represent 11 of the 12 mutations identified as causing florid CNS symptoms at the time these studies were conceived; to the best of our knowledge, these still represent about 80% of such mutations identified in the literature. In this study we compare the findings for those ten mutations to what we see for the four PNS-only mutations.

## Results

**Figure 1:**
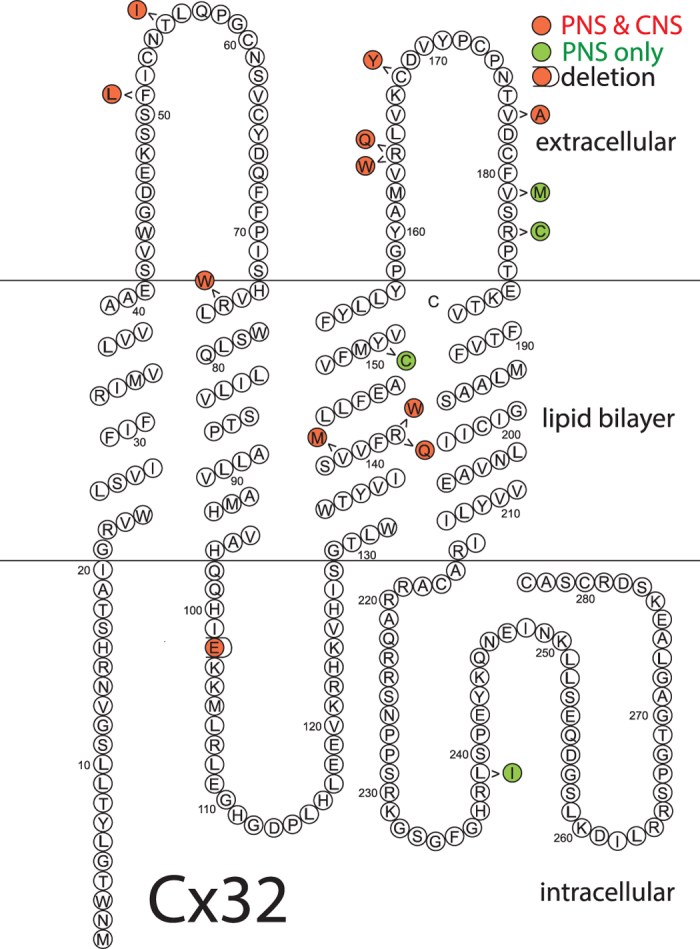
Graphic representation of the locations of the Cx32 mutations studied. Topology shown is based on Bennett and coworkers^59^. As shown, the PNS + CNS mutants are distributed throughout the length of Cx32 while all four PNS-only mutants are located in the C-terminal half of the protein.

The relevant clinical findings in the patients carrying the PNS + CNS mutations examined here and the corresponding literature references are given in Table 1. One other CNS mutant (R75W) previously studied by us^32^ is also included. In addition, we evaluated four mutants associated with typical CMT1X but lacking in CNS abnormalities; these patients had normal visual and brainstem auditory evoked potentials and no known episodes of acute, florid syndrome. Figure 1 shows the positions of the 14 Cx32 mutations studied. For the CNS + PNS mutants there are two in the first extracellular loop, four in the second extracellular loop and one in the second and three in the third transmembrane domains. Three of the PNS only mutants are located in the second extracellular loop while one is in the cytoplasmic domain.

### Plaque formation - Immunofluorescence Studies

To determine whether mutants can form plaques on the plasma membrane at regions of cell-cell contact, we examined the subcellular localization of all 15 Cx32 mutants. HeLa cells were transiently transfected to express either the WT Cx32 or one of the mutations. All mutants showed expression, indicating that none of the mutations affected immunoreactivity with our Cx32 antibody. Cells transfected with vector alone showed no immunoreactivity.

In every transfected cell, regardless of mutation, connexin expression could be confirmed by a substantial presence of immunoreactive protein in subcellular compartments within the cytoplasm, presumed to be the endoplasmic reticulum and Golgi apparatus, reflecting the normal pathway of Cx32 expression and oligomerization^33^. In addition to the intracellular immunoreactivity some cells exhibited plasma membrane staining. This typically shows as discontinuous fluorescent puncta along the membrane. Puncta are typically only seen where two cells are in direct contact and these have been shown to correspond to the gap junction plaques observed by electron microscope^34^. The possibility that functional gap junction coupling could be present in the absence of immunofluorescent puncta (i.e. without the channel clustering that produces bright immunofluorescent staining) is not supported by evidence from experiments in which EGFP-tagged connexin was used to correlate plaque size with the level of electrophysiologically measured coupling in live cells^35^,^36^. Therefore, only cells with punctate staining at the plasma membrane were deemed gap-junction competent.

**Figure 2:**
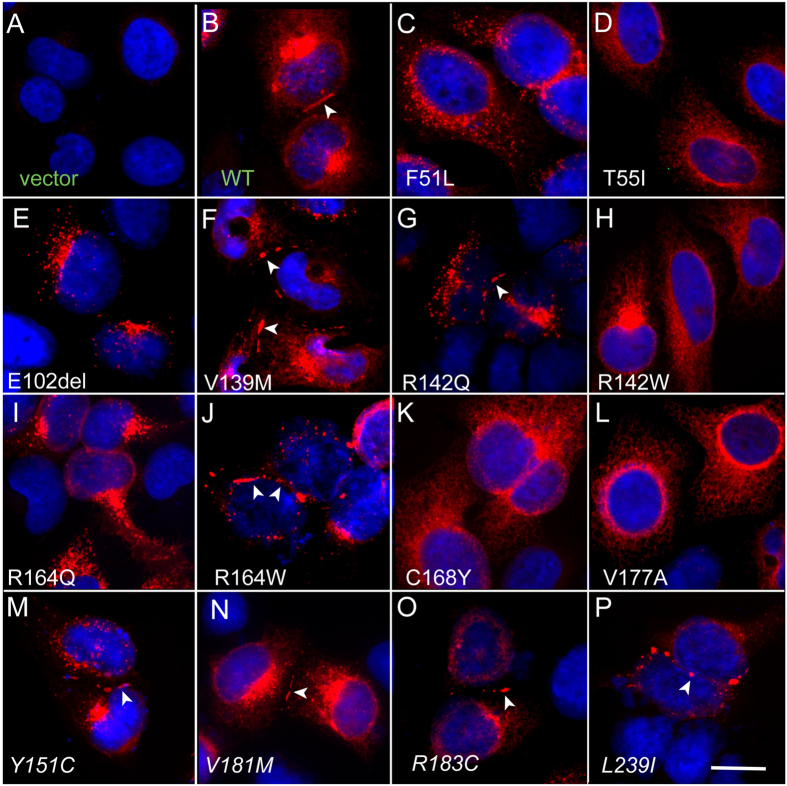
Connexin expression and plaque formation in HeLa cells transfected with WT and mutant Cx32. These are digital images of HeLa cells that have been transiently transfected to express one of the indicated mutations as well as empty vector (vector) and wild type Cx32 (WT). The cells were immuno-stained for Cx32 (red) and counterstained with DAPI (blue) to label nuclei. Some of the PNS + CNS mutants-F51L, T55I, E102G, R142W, R164W, C168Y and V177A - never displayed gap-junction plaques, while some - V139M, R142Q, and R164W – had gap junction plaques (arrows), as did all of the PNS-only mutants (*Y151C, V181M, R183C and L239I*) and WT Cx32. Scale bar = 10 microns.

In addition to the expected plaques observed in cells transfected with WT Cx32 we also found plaques in 7 of the 14 mutant Cx32-expressing cells – *Y151C, V181M, R183C, L239I*, R142Q, V139M, and R164W (Fig. 2). In the remaining 7 mutations (F51L, T155I, C168Y, R142W, E102del, R142Q, V177A and R164Q), we saw no evidence of plaque formation despite abundant intracellular fluorescence. Thus, the plaque-forming group is constituted by cells transfected with all four of the PNS-only mutations as well as three of the PNS + CNS mutations. All others appear to not traffic to the membrane to any significant extent and/or fail to dock with hemichannels on neighboring cells.

### Cell-cell coupling - Electrophysiological Studies

To further characterize differences between PNS-only and PNS + CNS mutants, we evaluated junctional conductance electrophysiologically in the 7 plaque-forming Cx32 mutants as well as WT and vector-only transfected cells. To evaluate the functional properties of each mutant, we transiently transfected them in Neuro2a cells and examined the conductance induced between pairs of these cells in response to voltage steps. This cell type is well suited for the dual whole-cell patch-clamp approach due its more spherical shape and compact size, which are attributes that enable effective voltage clamp throughout the cell. More importantly, Neuro2a cells are well-known for having virtually no contaminating endogenous connexin expression. Because the transfection vector expressed EGFP, transfected cells were selected for recording based on EGFP fluorescence. In this system, the presence of EGFP fluorescence provides a good marker for successful transfection^37^, as shown by the fact that presence or absence of fluorescence correlates with presence or absence, respectively, of gap junctional conductance when examining cells expressing Cx32WT, other WT connexins, or any number of mutants that form functional cell-cell channels. Therefore, using EGFP fluorescence to identify connexin-expressing cells, only pairs of cells in which both cells expressed EGFP were targeted for recording. Note that in these recordings both cells of a pair were expressing the same Cx32 variant (i.e. a homotypic configuration of cell-cell channel pairing).

**Figure 3:**
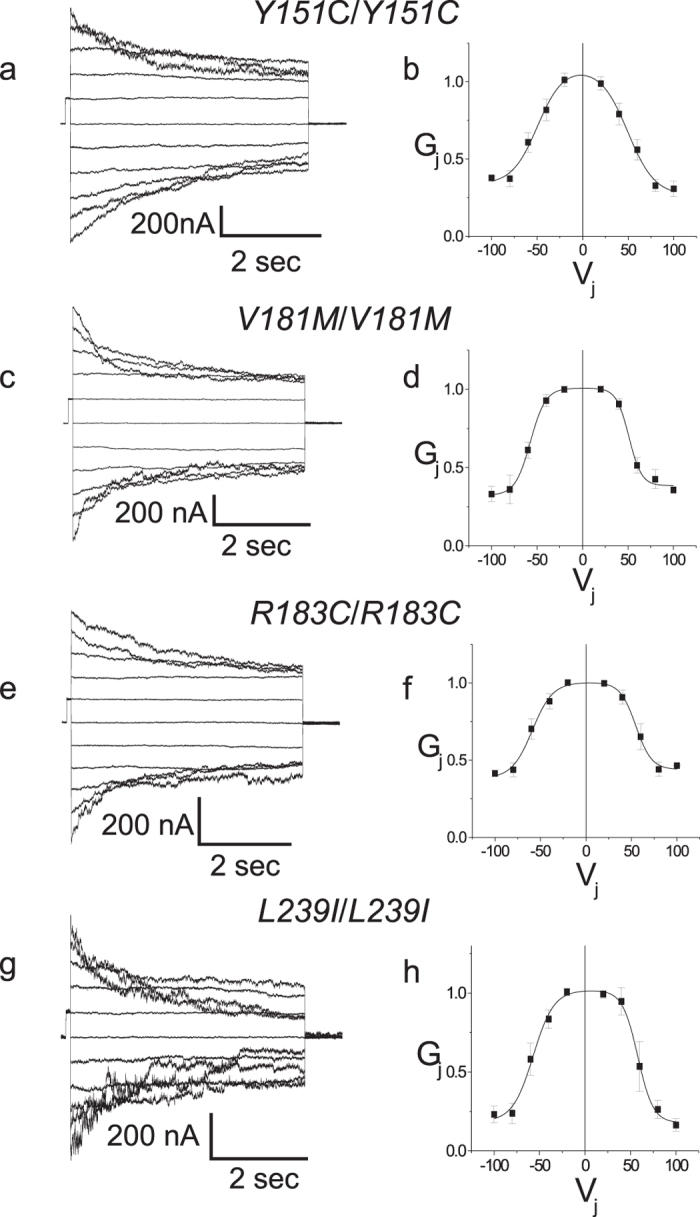
Homotypic pairing of PNS-only mutants of Cx32 reveals normal gap conductance properties. Macroscopic junctional currents for homotypically paired cells expressing the *Y151C* (**a**), *V181M* (**c**), *R183C* (**e**) and *L239I* (**g**) and the corresponding steady state G_j_ − V_j_ relations for homotypically paired cells (**b**,**d**,**f**,**h**, respectively). Mutant G_j_ − V_j_ relations are similar to those seen for Cx32WT though G_min_ differs in some mutants. In b, d, f, and h, data are shown as mean +/− SEM. Solid lines are Boltzmann fits to the data shown by the filled squares. See Table 3 for fit parameters.

**Figure 4:**
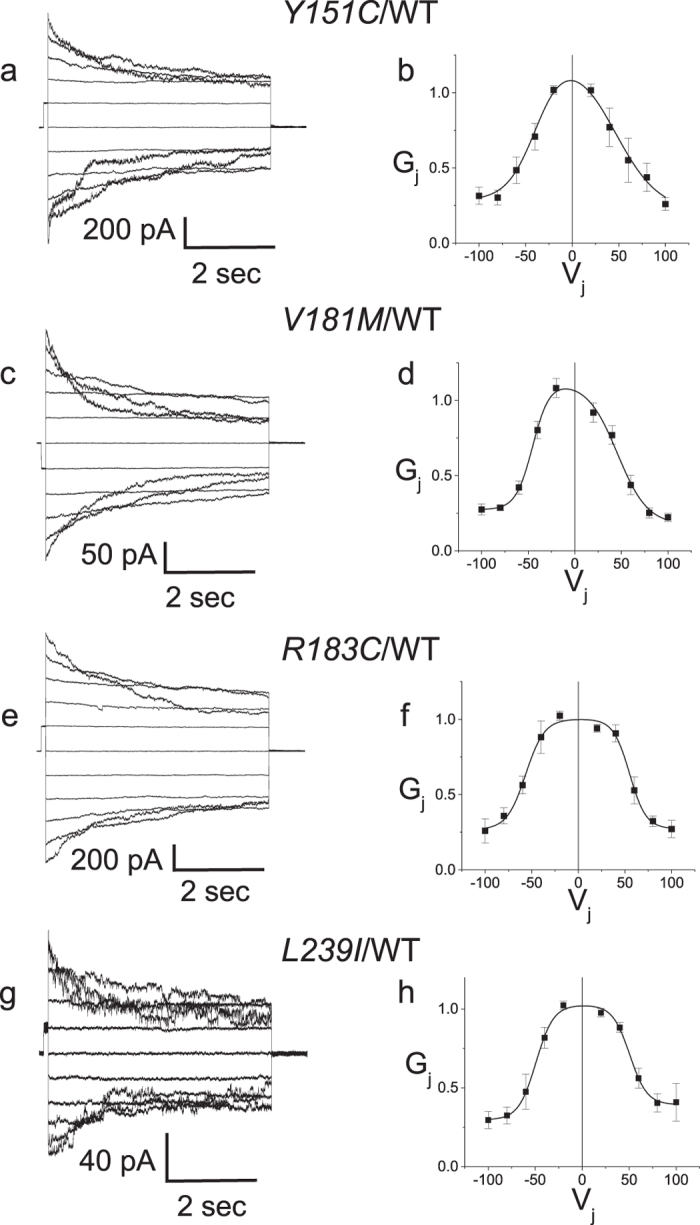
Heterotypic pairing of PNS-only mutants with WT Cx32 confirms normal gap junctional properties. Macroscopic junctional currents for heterotypically paired cells expressing the *Y151C* (**a**), *V181M* (**c**), *R183C* (**e**) and *L239I* (**g**) and the corresponding steady state G_j_ − V_j_ relations for homotypically paired cells (**b**,**d**,**f**,**h**, respectively). Mutants show G_j_ − V_j_ symmetry similar to that seen in homotypically paired Cx32WT. In (**b**,**d**,**f**,**h**), data are shown as mean +/− SEM. Solid lines are Boltzmann fits to the data shown by the filled squares. V_j_ is applied to the cell expressing the second connexin listed in the pairing configuration. See Table 3 for fit parameters.

Table 2 summarizes our findings on these 7 mutants. Briefly, all 4 PNS-only mutants (*Y151C, V181M, R183C, L239I*) exhibit a junctional conductance that is statistically indistinguishable from that recorded in cells transfected with WT Cx32. Figure 3 shows that all 4 PNS-only mutants also have current-voltage relations that are indistinguishable from that of WT Cx32. As shown in Table 3, this is also reflected in the similarity of the parameters for the Boltzmann fits to the data of Fig. 3 for Cx32 and the four PNS-only mutants. The similarity of the four mutants to Cx32WT is further demonstrated by heterotypic pairing of each mutant with Cx32WT (Fig. 4). In gap junctions, cell-cell channels are formed of two apposed hemichannels that dock together into a single cell-cell channel. *Homo*typic gap junction channels are composed of two identical apposed hemichannels. This creates a symmetric cell–cell channel and typically a symmetric conductance-voltage (G_j_ − V_j_) relation like those shown in Fig. 3, where in the simplest model each limb of the curve is due to gating of one of the two hemichannels. *Hetero*typic coupling, on the other hand, can produce symmetric or asymmetric G_j_ − V_j_ relations, depending on the similarity of properties of each of the two hemichannels being paired. The relatively symmetric Gj − Vj relations shown in Fig. 4 add further support to the notion that the four PNS-only mutants have normal functional properties.

In contrast to the PNS-only mutants, all three plaque-forming PNS + CNS mutants (R142Q, V139M and R164W) exhibited no junctional conductance in the homotypic configuration; i.e. results were statistically indistinguishable from what is observed in cells transfected with vector expressing EGFP only (Table 2). To confirm that the mutant protein of the 3 PNS + CNS mutants was indeed present at the plasma membrane, as suggested by the immunofluorescent puncta, we paired each of the 3 mutants with Cx32WT (heterotypic configuration).

**Figure 5:**
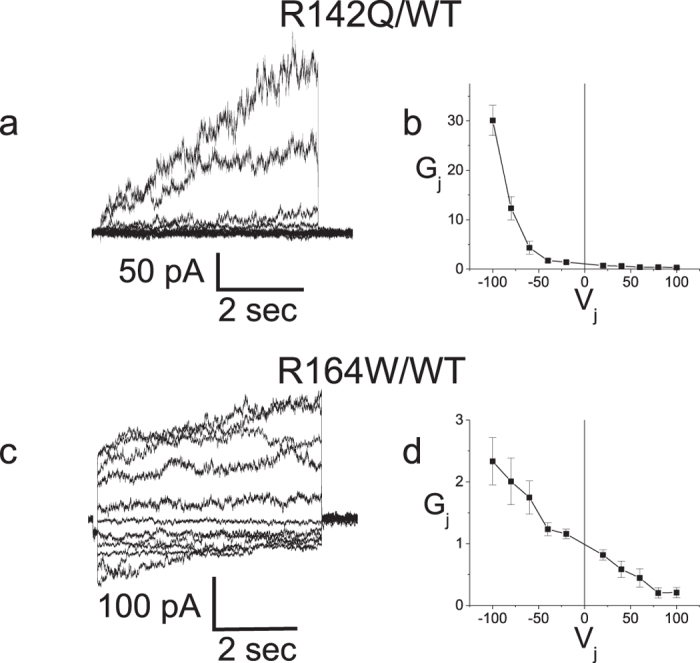
Heterotypic pairing of plaque-forming PNS + CNS mutants with WT Cx32 confirms the presence of two of the mutants at the plasma membrane. (**a**) Representative current traces recorded from an R142Q/Cx32WT pairing; Current responses were increasingly activated when negative pulses were applied to the Cx32 expressing cell. (**b**) Average normalized G_j_ − V_j_ relations for heterotypic R142Q/Cx32WT channels. (**c**) Representative current traces recorded from a R164W/Cx32WT pairing; Current responses were increasingly activated when negative pulses were applied to the Cx32 expressing cell. (**d**) Average normalized G_j_ − V_j_ relations for heterotypic R164W/Cx32WT channels. In (**a**) data was filtered at 500 Hz for display. In (**c**) data was filtered at 200 Hz for display. In (**b**,**d**) data are shown as mean +/− SEM. V_j_ is applied to the cell expressing the second connexin listed in the pairing configuration.

Unlike the homotypic configuration, which models the reflexive gap junctions between the layers of myelin sheath within a single myelinating Schwann cell (as well as the gap junctions formed between oligodendrocytes), the heterotypic configuration is likely to obtain *in vivo* only between oligodendrocytes in the CNS of female carriers, where a Lyonized pattern of expression can occur. In this experiment we used it primarily as a way of detecting the presence of the mutant connexin on one side of the gap junction (i.e. its presence at the plaque) by its effect on the conductance of the WT channels on the other cell. For two of the PNS + CNS mutants (R142Q and R164W), we were able to demonstrate that the mutant was present at the gap junction by virtue of its ability to generate an asymmetrical conductance-voltage (G_j_ − V_j_) relation (Fig. 5). This type of asymmetrical Gj − Vj relation is never observed in Cx32WT-expressing cells paired with cells transfected with vector-EGFP (or vector-IRES DsRed), a situation in which no conductance is seen at all. However, to capitalize on heterotypic coupling to confirm the presence of the mutant at the membrane the mutant needs to exhibit an open state at *some* transjunctional voltage, irrespective of how non-physiological this voltage may be. One also relies on the ability of mutant hemichannels in one cell to dock with the WT hemichannels in the other to form a cell-cell channel. For the third mutant (V139M), all we could detect was a small amount of very asymmetric junctional current in one of ten cell pairs. A likely explanation is that V139M hemichannels are able to dock between cells but have an extremely low probability of exhibiting the open state. (In addition to using EGFP as a marker for transfection, Cx expression in N2a cells was confirmed for this mutant using immunocytochemistry; not shown.) This is consistent with work from our laboratory^38^ showing that in oocytes the V139M mutant formed functional heterotypic channels that showed a very low open probability at relevant voltages. Together, our results make it highly unlikely that any of the PNS + CNS mutants can form functional gap junctions *in vivo* even when exhibiting gap junction plaques on immunofluorescent studies.

## Discussion

Over 400 mutations in Cx32 have been identified in patients with CMT1X (http://hihg.med.miami.edu/code/http/cmt/public_html/index.html#/); for most, no information is available on whether they could lead to acute florid CNS dysfunction. Many CMT1X patients have stable subclinical abnormalities in their central nervous system as evidenced by abnormal (but asymptomatic) visual or auditory evoked responses. For this reason, we decided to restrict our definition of “PNS-only” mutants to only those which, in addition to having had no history of episodic CNS symptoms, were documented to have shown no evidence of overt or even subclinical CNS involvement, including documentation of normal visual and auditory evoked responses. With this definition as the basis for establishing a potential correlation between symptomatology and the nature of connexin dysfunction we first examined the subcellular localization of ten CMT1X mutations of Cx32 associated with acute transient neurologic CNS dysfunction as well as the only four mutants satisfying our PNS-only definition.

There is strong justification for using the presence of punctate staining as a necessary (although not sufficient) condition for a mutant’s ability to form functional gap junctions. This is because whenever WT connexins are examined, there is a one-to-one correlation between punctate staining and functional gap junctions^35^,^39^,^40^,^41^. In the few cases in which a connexin does not form GJs (e.g. Cx29^42^) punctate staining is also not found^43^. When studying subcellular localization of connexins, the ability of connexin mutants to exhibit plaques in HeLa cells has uniformly been a good predictor of plaque formation ability *in vivo*, both in terms of failure to form plaques (T55I^44^,^45^, R75W^45^,^46^, R142W^47^ (and this report) and in terms of successful plaque-formation (280 G^44^,^48^). Therefore, in this study we used plaque formation in HeLa cells as a screen for the GJ-forming potential of each mutant examined. In the second part of the study we used Neuro2a cells for exogenous expression of all 7 CMT1X mutants that passed the plaque formation screen. Neuro2a cells are a useful model for studying the junctional properties of connexins, both because of their extremely low levels of endogenous coupling and because they are generally well suited for patch clamp electrophysiological recording.

While the number of clinically-characterized PNS-only mutations is not as high as one would need to make more definitive statements, several trends do emerge from our data which support a distinction between PNS-only versus PNS + CNS based on parameters related to junctional conductance. First, all four PNS-only mutants produce gap junction plaques as we show in HeLa cells. In addition, all four PNS-only mutants show steady state conductance-voltage relations and magnitudes of junctional coupling that differ only subtly from that of WT Cx32. A number of other mutant forms of Cx32 have similarly been demonstrated to show relatively normal conductance voltage relations. and/or normal levels of coupling in oocytes^4^,^49^,^50^,^51^. However only a few have been evaluated in mammalian cells for both magnitude of coupling and conductance voltage characteristics and none of these mutations have been reported in patients with florid acute syndromes, the focus of this study. Second, all PNS + CNS mutants studied, including the previously studied R75W mutant, are either unable to form gap junction plaques or have such abnormal conductance characteristics as to render them functionally unable to mediate coupling at the range of transjunctional voltages likely to obtain between glial cells. In other words, PNS + CNS mutants are associated with changes predicted to lead to complete loss of function of homotypic Cx32 channels.

Our results, when viewed in the context of the known clinical data, suggest the possibility that retention of normal or near normal cell-cell junctions may protect against florid CNS symptoms and possibly against slowing of central conductions (i.e. abnormal VERs and BAERs). In other words, disruption of this function could be a necessary condition for florid CNS symptoms. This line of reasoning is supported by the recent report of recurrent florid symptoms in a patient carrying a Met1Ile mutation^28^, predicted to lead to complete loss of Cx32 protein^52^. This reasoning also suggests the possibility that complete loss of reflexive coupling in Schwann cells may lead to reduced peripheral nerve conduction velocities in patients with CNS + PNS mutants when compared to those with PNS only mutants, a possibility that could potentially be clinically assessed in the future. At the same time, the episodic nature of florid symptoms also suggests that additional not yet identified genetic and/or environmental factors are necessary for CNS manifestations in CMT1X patients. Moreover, if these factors were to have different effects depending on the mutation expressed this would add further diversity to the pathogenesis of CNS symptoms in CMT1X.

In the CNS, oligodendrocyte-oligodendrocyte (O:O) coupling is mediated by Cx32:Cx32 and Cx47:Cx47 homotypic channels, and oligodendrocyte-astrocyte (O:A) coupling is mediated by Cx32:Cx30 and Cx47:Cx43 heterotypic channels^5^. Thus, it is likely that CNS Cx32 mutations affect both Cx32:Cx32 (O:O) and Cx32:Cx30 (O:A) coupling, However, it appears that disrupted Cx32:Cx32 (O:O) coupling is likely to be the more important for the transient CNS dysfunction because the lesions are in the white matter where, at least in mice, O:O coupling predominates over O:A coupling^6^,^7^. Our results suggest that in CNS, loss of junctional coupling through Cx32 only becomes an important factor under specific but yet to be defined circumstances. Moreover, in light of emerging evidence that connexins play roles other than their classical gap junction-forming role the less predictable nature of CNS manifestations may reflect other, non- junctional, roles for Cx32 in oligodendrocytes.

In the PNS, our data suggest that the ability of Cx32 to form gap junction channels may not even be a deciding factor in whether a mutation causes disease. Nonetheless, clinical data suggest that loss of function is sufficient to cause PNS disease, since patients with deletion of the entire coding sequence of GJB1 or nonsense mutations near the N terminus have a PNS phenotype very similar to that seen in patients with other mutations^53^. It is possible that, whereas conductance of small electrolytes appears to not be significantly affected in the PNS-only mutants, the flux of some larger biologically important molecule(s) could be affected as shown previously for the S26L^54^,^55^, Delta111–116^54^ and R220stop^54^ mutants. Such a critical molecule, if one exists, remains to be identified. However, one candidate is ATP, the release of which appears to be dependent on Cx32 hemichannels and is likely affected by mutations in GJB1^56^. In this regard, this study has shown that the four PNS-only mutations, if they indeed obstruct the flux of larger molecules, could be useful in future studies aimed at determining which small molecules could be relevant to CMT1X pathology in peripheral nerve. At the same time, the notion that the primary role of Cx32 in Schwann cells is to mediate a radial pathway for diffusion through reflexive junctional coupling in non-compact myelin (its only proposed role in Schwann cells) might need to be reconsidered, as previously suggested by *ex vivo* experiments in which dye tracers demonstrated the presence of a reflexive pathway in the peripheral nerve of both Cx32-expressing and Cx32-lacking mice^57^.

## Methods

### Analysis of transfected cells-immunofluorescent staining

Mutants were generated using the QuikChange II XL Site-Directed Mutagenesis Kit. (Stratagene, La Jolla, CA), from a human *GJB1* cloned into pIRES2-EGFP or pIRESpuro3 and subcloned as needed into pIRES2-EGFP (Clontech, Mountain View, CA)^5^; mutations were confirmed by sequencing.

Communication-incompetent HeLa cells originally obtained from Prof. Klaus Willecke (Institute for Genetics, University of Bonn, Germany) were grown to 80% confluency. Transient transfection utilized Jetprime (Polypus Transfection, Illkirch, France) and DNA containing either WT Cx32, or the relevant Cx32 mutations according to manufacturer’s instructions. 28 hours later, cells were washed in phosphate buffered saline (PBS), fixed in acetone at −20 °C for 10 min, and blocked with fish gelatin in PBS containing 0.1% Triton for 1 hr. at room temperature (RT). Primary antibodies were added in the same blocking solution and the samples incubated overnight at 4 °C. For Cx32 staining, a rabbit antiserum to the C terminus produced in the Scherer lab^58^ was used (diluted 1:2500). After washing in PBS, fluorescein-conjugated donkey cross-affinity purified secondary antibodies (diluted 1:200; Jackson ImmunoResearch, West Grove, PA) were added in the same blocking solution for 1 hr at RT. Cell nuclei were visualized with 4′,6′-diamidino-2-phenylindole (DAPI) (Sigma). Slides were covered with mounting medium (Vectashield, Vector Laboratories, Burlingame, CA) and images were photographed under a Leica epifluorescence microscope with a digital camera.

### Analysis of transfected cells-dual whole cell recordings

pIRES2-EGFP vectors containing DNA encoding human Cx32WT or mutants were transiently transfected into confluent Neuro2a cells. Homotypic cell pairs were prepared by re-plating one day following transfection. For heterotypic cell pairs, transfected cells were washed and cells expressing a pIRES2-EGFP construct expressing mutant Cx32 were mixed with cells expressing a pIRES2-DsRed construct expressing WT Cx32, in a 1:1 ratio. Coupling was assessed by dual whole-cell patch clamping of cell pairs 6–48 hours after re-plating as previously described^32^. Only cells clearly expressing the appropriate fluorescent proteins (thus indicating that they were transfected) were used for recording. Recording solutions used were as follows (in mM): pipette solution, 145 CsCl, 5 EGTA, 1.4 CaCl_2_, and 5.0 HEPES, pH 7.2; bath solution, 150 NaCl, 4 KCl, 1 MgCl_2_, 2 CaCl_2_, 5 dextrose, 2 pyruvate, and 10 HEPES, pH 7.4. Heterotypic pairings between cells are shown as “connexin expressed in cell 2/connexin expressed in cell 1”; normalized junctional conductance-junctional voltage (G_j_ − V_j_) relations were determined from isolated pairs by measuring junctional current (I_j_) responses in cell 2 following 12.5 second junctional voltage pulses (from −100 mV to 100 mV in 20 mV increments) applied to cell 1, and applying Ohm’s law. For all homotypic and heterotypic pairings with G_j_ − V_j_ plots fit to a double Boltzmann distribution, steady state conductance levels were estimated by fitting current traces to a sum of exponentials and using the conductance level at t = ∞ Baseline (V_j_ = 0) junctional conductances were similarly determined by measuring instantaneous I_j_ responses to voltage steps from V_j_ = 0 to +40 mV and to −40 mV and using the average of the two measurements. Cytoplasmic bridges were excluded by demonstrating sensitivity of junctional conductance to application of octanol-containing bath solution. G_j_’s were not corrected for series resistance. For G_j_ − V_j_ plots, data was imported into Origin (Originlab, Northampton, CA), and fit to a “double Boltzmann” equation of the form:


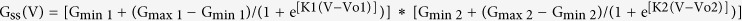


where G_ss_ is the steady state junctional conductance normalized to V_j_ = 0, G_max1_ and G_max2_ are the maximal and G_min1_ and G_min2_ the minimal normalized conductances for the limb reflecting the normalized residual conductances for the negative or positive limbs, and V_01_ and V_02_ are the voltage at which the conductance for the negative or positive limb is 1/2 of the difference between G_min_ and G_max_ for that limb. K_1_ and K_2_ are parameters which reflect the slope of the negative or positive limb of the G_j_ − V_j_ plot and are a measure of voltage sensitivity. See Abrams *et al*.^49^ for further discussion.

### Statistics

All statistical tests were performed in GraphPad (San Diego, CA) Prism. For dual whole-cell patch clamp assays junctional conductance values were compared using the Kruskal-Wallis test with Dunn’s post-test.

## Additional Information

**How to cite this article**: Abrams, C. K. *et al*. Loss of Coupling Distinguishes GJB1 Mutations Associated with CNS Manifestations of CMT1X from Those Without CNS Manifestations. *Sci. Rep.* **7**, 40166; doi: 10.1038/srep40166 (2017).

**Publisher's note:** Springer Nature remains neutral with regard to jurisdictional claims in published maps and institutional affiliations.

## Figures and Tables

**Table 1:** Summary of the CNS clinical phenotypes of patients with mutations studied in this communication.

Mutant	Trigger	Clinical	References
F51L	Returning from high altitude	ADEM-like illness, with MRI changes and full recovery.	Scherer, unpublished
T55I	None noted	Two brothers with transient CNS generalized weakness, dysarthria, dysphagia, and MRI changes. An unrelated 63 yo patient had an abnormal MRI.	15,16
R75W	4 unprovoked; worst attack after wrestling	Two sets of attacks − 3 closely-spaced attacks at 12 yo; one unprovoked attack followed by one provoked attack at 15 yo, with MRI changes.	19
E102del	Mother – infections, Son 1 - exercise, and after return from mountains, Son 2 - infection surgery	Mother’s symptoms recurred at irregular intervals. Son 1: tetraparesis for 1 hour after football practice; confusion, weakness for 3 hours, weeks after being at high altitude. Son 2: multiple brief, 1 hour episodes during childhood. One severe episode at 19 yo, following appendectomy in setting of mononucleosis. Both showed MRI WM changes.	20
V139M	Fever, Trauma?, Infection?	Yearly < 1 hour episodes after fever. Longer, 12 hour episode at age 17 with relapse on day 2. 2 brothers: 13 yo with 1/2 day episode 2 weeks after concussion with relapse a day later; 16 yo with hemiparesis and WM changes on MRI, 2 weeks after pneumonia.	29,22
R142Q		Transient neurological findings and WM changes on MRI. Associated with deafness.	S. Yum PC^60^
R142W	Return from high altitudes	26 yo with subacute dysarthria, incoordination, difficulty walking, and a feeling of weakness within days of returning from alt of 8000 ft. lasting 2 weeks but waxing and waning. 41 yo female patient with WM changes on MRI.	17,13
R164Q	Fever, Post influenza infection?	14 yo with 3 days paresthesia, disorientation, ataxia and weakness and foci of WM changes on MRI.	26
R164W	Fever?None noted	14 yo with 6 hours of headache, nausea, vertigo, and fever. bilateral facial weakness, dysarthria, dysphagia, pyramidal right-sided weakness. 44 yo man with 10 y of CNS symptoms; normal VERs, abnormal BAERs, MRI findings; no clinical event for CNS involvement. 13 yo with 30 minutes of slurred speech and leg weakness followed a week later by 36 hours of right hemiparesis and dysphasia and diffuse WM changes on MRI.	18,21,30
C168Y	return from high altitudes	16 yo with 5 days of multiple 8-10 hr episodes with tingling in the fingers of both hands and the perioral region, progressing to severe dysarthria, weakness, and ataxia and WM changes on MRI.	17
V177A		7 yo with 36 hours of left hemiparesis and dysphasia.	24
*Y151C*		PNS-only; no evoked potential abnormalities.	ref. 31 and PC G. Nicholson
*V181M*		PNS-only; no evoked potential abnormalities.	ref. 31 and PC G. Nicholson
*R183C*		PNS-only; no evoked potential abnormalities.	ref. 31 and PC G. Nicholson
*L239I*		PNS-only; no evoked potential abnormalities.	ref. 31 and PC G. Nicholson

WM: white matter; yo: year old; ADEM: acute disseminated encephalomyelitis; PC: personal communication.

**Table 2:** Summary of coupling between pairs of cells expressing the indicated mutants, WT Cx32 or vector-EGFP in the homotypic configuration.

	n	Mean	SD	SE	p vs ie	p vs 32wt	
V139M	8	0.23	0.57	0.20	NS	*	CNS + PNS
R142Q	6	0.09	0.20	0.08	NS	**	CNS + PNS
R164W	16	0.05	0.12	0.03	NS	***	CNS + PNS
*Y151C*	8	20.99	17.92	6.34	***	NS	PNS only
*V181M*	7	50.24	42.78	16.17	**	NS	PNS only
*R183C*	15	30.57	44.41	11.47	*	NS	PNS only
*L239I*	12	27.37	37.63	10.86	**	NS	PNS only
32WT	21	51.76	57.49	12.55	***		
ie	16	0.30	1.12	0.28		***	

ie: empty pIRES2-EGFP vector; SD: standard deviation; SE: standard error of the mean; *p < 0.05; **: p < 0.01; ***p < 0.001;NS not significant.

**Table 3:** Summary of Boltzmann parameters for Cx32WT and PNS-only mutants for homotypic and heterotypic pairings.

	G_max_ ( + V)	G_min_ ( + V)	V_0_ ( + V)	K ( + V)	G_max_ (−V)	G_min_ (−V)	V_0_ (−V)	K (−V)
Homotypic								
32WT	1.02	0.22	49.37	10.21	1.04	0.21	−48.06	−10.65
Y151C	1.08	0.26	49.43	14.94	1.02	0.31	−49.55	−13.97
V181M	1.04	0.30	55.59	10.98	0.99	0.25	−54.71	−10.14
R183C	0.92	0.40	54.86	8.68	1.09	0.42	−59.01	−11.24
L239I	1.00	0.18	57.97	8.46	1.02	0.19	−57.04	−11.31
Heterotypic								
Y151C/32WT	1.05	0.24	52.38	14.55	1.04	0.3	−44.36	−16.81
V181M/32WT	1.06	0.26	45.73	16.19	1.05	0.16	−45.20	−8.99
R183C/32WT	1	0.27	54.97	8.75	1	0.27	−56.58	−10.06
L239I/32WT	0.95	0.28	50.68	9.12	1.08	0.41	−49.21	−9.25
